# The Accuracy of Zygomatic Implant Placement Assisted by Dynamic Computer-Aided Surgery: A Systematic Review and Meta-Analysis

**DOI:** 10.3390/jcm12165418

**Published:** 2023-08-21

**Authors:** Shengchi Fan, Gustavo Sáenz-Ravello, Leonardo Diaz, Yiqun Wu, Rubén Davó, Feng Wang, Marko Magic, Bilal Al-Nawas, Peer W. Kämmerer

**Affiliations:** 1 Department of Oral and Maxillofacial Surgery, Plastic Operations, University Medical Center Mainz, 55131 Mainz, Germany; 2 Second Dental Clinic, Ninth People’s Hospital, College of Stomatology, Shanghai Jiao Tong University, School of Medicine, National Clinical Research Center for Oral Disease, Shanghai Key Laboratory of Stomatology, Shanghai Research Institute of Stomatology, Shanghai 200011, China; 3 Center for Epidemiology and Surveillance of Oral Diseases (CESOD), Faculty of Dentistry, Universidad de Chile, Santiago 8380420, Chile; 4 Postgraduate School, Faculty of Dentistry, Universidad de Chile, Santiago 8380420, Chile; 5 Department of Implantology and Maxillofacial Surgery, Vithas Davó Instituto Dental, 03016 Alicante, Spain; 6 School of Dental Medicine, University of Belgrade, 11000 Belgrade, Serbia

**Keywords:** zygomatic implant, edentulous, computer-aided surgery, navigation, guided surgery

## Abstract

Purpose: The present systematic review aimed to investigate the accuracy of zygomatic implant (ZI) placement using dynamic computer-aided surgery (d-CAIS), static computer-aided surgery (s-CAIS), and a free-hand approach in patients with severe atrophic edentulous maxilla and/or deficient maxilla. Methods: Electronic and manual literature searches until May 2023 were performed in the PubMed/Medline, Scopus, Cochrane Library, and Web of Science databases. Clinical trials and cadaver studies were selected. The primary outcome was planned/placed deviation. Secondary outcomes were to evaluate the survival of ZI and surgical complications. Random-effects meta-analyses were conducted and meta-regression was utilized to compare fiducial registration amounts for d-CAIS and the different designs of s-CAIS. Results: A total of 14 studies with 511 ZIs were included (Nobel Biocare: 274, Southern Implant: 42, SIN Implant: 16, non-mentioned: 179). The pooled mean ZI deviations from the d-CAIS group were 1.81 mm (95% CI: 1.34–2.29) at the entry point and 2.95 mm (95% CI: 1.66–4.24) at the apex point, and angular deviations were 3.49 degrees (95% CI: 2.04–4.93). The pooled mean ZI deviations from the s-CAIS group were 1.19 mm (95% CI: 0.83–1.54) at the entry point and 1.80 mm (95% CI: 1.10–2.50) at the apex point, and angular deviations were 2.15 degrees (95% CI: 1.43–2.88). The pooled mean ZI deviations from the free-hand group were 2.04 mm (95% CI: 1.69–2.39) at the entry point and 3.23 mm (95% CI: 2.34–4.12) at the apex point, and angular deviations were 4.92 degrees (95% CI: 3.86–5.98). There was strong evidence of differences in the average entry, apex, and angular deviation between the navigation, surgical guide, and free-hand groups (*p* < 0.01). A significant inverse correlation was observed between the number of fiducial screws and the planned/placed deviation regarding entry, apex, and angular measurements. Conclusion: Using d-CAIS and modified s-CAIS for ZI surgery has shown clinically acceptable outcomes regarding average entry, apex, and angular deviations. The maximal deviation values were predominantly observed in the conventional s-CAIS. Surgeons should be mindful of potential deviations and complications regardless of the decision making in different guide approaches.

## 1. Introduction

The zygomatic implant (ZI) was introduced for the oral rehabilitation of patients who had undergone maxillectomy and became an alternative treatment to avoid extensive bone grafting for edentulous atrophic maxillae [1]. The use of ZI for the rehabilitation of maxillary defects and edentulous maxillae provides predictable long-term survival and significantly improves patient quality of life [2,3]. The classic approach is indicated for patients without sufficient bone height in the posterior area. It involves placing one ZI in the molar/premolar site, combined with 2 to 4 dental implants [1]. Subsequently, the quad zygoma approach has been applied for patients with a complete absence of bone in the anterior and posterior regions [4]. In this approach, two ZIs are well distributed on each side of the alveolar process and zygoma. Due to the benefit of anchoring the implant in zygomatic bone, patients can carry a functional prosthesis immediately after the ZI surgery [5].

However, due to the anatomical intricacies of zygomatic processes and limited intra-operative visibility, free-hand osteotomy of ZI often presents a significant challenge, especially for inserting multiple ZIs on one side of the zygoma with little experience. A recent systematic review and a retrospective study have reported various complications, including facial hematoma, lip laceration, malposition, orbital penetration, and zygoma bone fracture, all related to the operation [6,7]. Chrcanovic et al. demonstrated that conventional surgical guides could not provide accurate placement at the level of zygomatic bone [8]. Due to the extra-long trajectory, even a small amount of drilling mobility at the entry point can result in a significant deviation in the apex point within the zygomatic bone.

In 2016, a modified surgical guide with double sleeves, designed to stabilize the long drill and to overcome the deviation, was introduced [9]. Later, studies focused on utilizing this modified guide concept to assist in ZI placement with increased intra-operative safety and accuracy [10,11,12,13,14,15]. Gecchi et al. demonstrated that the accuracy of ZI placement with a modified surgical guide was superior to the free-hand approach, particularly at the level of the zygomatic bone [10]. Vosselman et al. successfully used a revised 3D-printed guide to assist in ZI placement in the patient with unilateral maxillary defect [13].

Real-time surgical navigation systems have been developed and applied to optimize pre-operative planning and provide constant trajectory visualization during implant insertion [16]. In 2000, Watzinger et al. performed the first cadaver study of navigation system-assisted ZI placement [17]. With the help of dynamic computer-aided guidance, ZI placement has become more accurate while providing the advantage of implementing the restorative plan into the implant position [18]. In a recent study, 221 ZIs were placed using real-time navigation system in 71 patients by classic or quad zygoma approach, as well as in patients with maxillary defects. The results showed high accuracy and high survival rates with a mean 2-year follow-up [19].

Different concepts and protocols have been proposed in recent years to increase the accuracy of ZI placement with computer-aided guidance [20,21,22,23]. However, most of these studies still need strong evidence, relying on randomized clinical trials instead on case reports, technical notes, or experimental model studies. The most recent systematic review, conducted in 2020, attempted to evaluate the data on navigation system-assisted ZI placement [24]. However, this review needed larger sample size and there was a lack of investigation related explicitly to navigation. Therefore, the primary aim of the present systematic review was to investigate the accuracy of ZI placement using dynamic computer-aided surgery (d-CAIS), static computer-aided surgery (s-CAIS), and the free-hand approach in patients with severely atrophic edentulous maxilla and/or deficient maxilla. The secondary purpose was to evaluate the survival of ZI and surgical complications associated with using different guidance techniques.

## 2. Materials and Methods

### 2.1. Prospero Registration

This systematic review was conducted with the Cochrane Collaboration guidelines and followed the Transparent Reporting of Systematic Reviews and Meta-Analyses (PRISMA) guidelines [25]. The review protocol was specified and registered at PROSPERO (International prospective register of systematic reviews) and received registration number: CRD42023431143.

### 2.2. PICOs Question

P: patient presented with a severely atrophic maxilla and/or maxillary defect;

I: ZI assisted with d-CAIS;

C: ZI placed free-hand or assisted with s-CAIS or none;

O_1_: measurements of planned/placed deviation;

O_2_: survival and complications.

### 2.3. Eligibility Criteria

Articles that met the following criteria were included in this systematic review: (1) all primary studies, including clinical (i.e., randomized clinical trials (RCTs), prospective and retrospective cohort studies, case–control studies, and case series with at least three patients), “human”, and “cadaver” studies that reported the accuracy of free-hand, static and dynamic computer–aided surgery of ZI; (2) studies reporting accuracy as the primary outcome; and (3) studies reporting the exact deviation between the pre-surgical planning and the final position of the ZI.

Therefore, the exclusion criteria were: (1) case reports and studies only assessing virtual, augmented reality, or virtual placement, and (2) studies focused on technical descriptions or lacking measurable clinical outcomes. (3) In vitro/model studies were excluded as the direct visualization cannot reflect the clinical situation.

### 2.4. Information Sources and Search Strategy

Electronic and manual literature searches until May 2023, conducted by two independent reviewers (LD and GS), were performed using PubMed/Medline, Scopus, Cochrane Library, and Web of Science. The search was started in PubMed and completed with Scopus, Cochrane Library, and Web of Science. A search string was created combining Boolean operators “AND” and “OR” and the following search terms/MeSH/keywords: (“computer-assisted surgery” [MeSH Terms] OR “guided surgery” (All Fields) OR “navigation surgery” (All Fields) OR “navigation system” (All Fields) OR “navigation systems” (All Fields) OR “real-time system” (All Fields) OR “real-time navigation” (All Fields) OR “dynamic guided surgery” (All Fields) OR “dynamic computer aided” (All Fields) OR “dynamic computer assisted” (All Fields) OR “computer-aided surgery” (All Fields) OR “freehand” (All Fields) AND “zygomatic implant” (All Fields) OR “zygomatic implants” (All Fields) OR “zygoma implant” (All Fields) OR “zygoma implants” (All Fields) OR “zygomatic fixture” (All Fields) AND “accuracy” (All Fields)). Additionally, a manual search was performed in the reference lists of selected articles, in Google Scholar and all issues since 2010 of the following journals: Annals of Maxillofacial Surgery, British Journal of Oral and Maxillofacial Surgery, Clinical Implant Dentistry and Related Research, Clinical Oral Implants Research, Dental Clinics of North America, European Journal of Oral Implantology, European journal of prosthodontics and restorative dentistry, Implant Dentistry, Imaging Science in Dentistry, International Journal of Implant Dentistry, International Journal of Oral and Maxillofacial Implants, International Journal of Oral and Maxillofacial Surgery, International Journal of Oral Implantology, International Journal of Periodontics and Restorative Dentistry, International Journal of Prosthodontics, Journal of Advance Prosthodontics, Journal of Clinical Periodontology, Journal of Cranio-Maxillo-Facial Surgery, Journal of Oral and Maxillofacial Surgery, Journal of Oral Implantology, Journal of Periodontal and Implant Science, Journal of Periodontal Research, Journal of Periodontology, Journal of Prosthetic Dentistry, Journal of Prosthodontic Research, Journal of Prosthodontics, Journal of Stomatology, Oral and Maxillofacial Surgery, Journal of the Korean Association of Oral and Maxillofacial Surgeons, Oral and Implantology, Oral and Maxillofacial Surgery, Oral and Maxillofacial Surgery Clinics of North America, Oral Surgery Oral Medicine Oral Pathology Oral Radiology and Endodontology, and Periodontology 2000. The languages of the studies included in the search were English and Spanish. Finally, previous systematic reviews investigating implant navigation surgery were also screened to identify potential articles. Search queries are available in Table S1 (Supplementary Materials). Cohen’s Kappa test was computed in Microsoft Excel 2022 (Microsoft Corporation, Redmond, OR, USA) to assess inter-rater agreement for two reviewers. Inter-rater agreement was interpreted according to the categories proposed by Landis and Koch [26].

### 2.5. Data Extraction

Two reviewers (SCF, RD) checked the primary studies against the PICO question of this review and then independently extracted their data, which two peer reviewers (YQW, FW) subsequently verified.

A summary of study characteristics included author(s), year of publication, study type, guide approach and system, number of samples, number of ZI, surgical approach (classic/quad/maxillary defect), outcomes measured, conclusion, indication, survival rate, complication, and implant placement errors means and standard deviations (SDs; Table 1, Table 2 and Table 3). The corresponding author was contacted to clarify any missing or unclear information in the included studies.

### 2.6. Risk of Bias

Two examiners (GSR and SCF) conducted the risk of bias assessment. Cochrane RoB 2 tool was used for RCT [27]. Non-randomized intervention studies were assessed using the ROBINS-I (Risk of Bias in Non-randomized Studies—of Intervention) [28]. Using the tools described above allows for a better comparison of evidence from RCTs and non-randomized studies because they sit on a common risk of bias metric [29].

### 2.7. Data Synthesis

A qualitative and quantitative synthesis of the included studies was performed. When possible, patient-level data were combined following the recommendations of the Cochrane Collaboration. The quantitative synthesis involved pooling the results from subsamples within the studies. Given the expected diversity between the studies (clinical heterogeneity), a single-mean random-effects meta-analysis using restricted maximum likelihood was chosen to pool the study results. The analysis produced a relative measure expressed as an overall mean and its corresponding 95% confidence interval (CI). Since no comparative studies were identified in the previous searches, an indirect comparison between d-CAIS, s-CAIS, and free-hand approaches was conducted using the chi-square test (*p* < 0.05). Heterogeneity was assessed using the chi-squared test (*p* < 0.1), and the I^2^ test was used to determine the proportion of variability attributed to between-study heterogeneity. A subgroup analysis was conducted based on the ZI protocol, specifically classifying studies as classic, quad zygoma, or maxillary defects approaches. The outcomes were presented according to the subgroup analysis or as a measure of the overall effect (*p* < 0.1 or *p* > 0.1 from the test for subgroup differences, respectively).

Mixed-effects meta-regression using the Hartung–Knapp method for random-effects meta-analysis was performed to assess the approximate number of screws used to register the position in the navigation system and the type of surgical guide as independent variables for the outcomes (*p* < 0.05) [30]. Publication bias was investigated for each outcome by visual inspection of the asymmetries in the funnel plot. If possible (having at least six studies), a statistical assessment of publication bias was performed using Egger’s test for funnel plot symmetry (*p* < 0.1) [31]. In addition, a sensitivity analysis was conducted to assess the impact of risk of bias (low to high) and type of study (cadaveric vs. clinical) on the effect estimates. Statistical analysis was performed by an experienced reviewer (GSR) using the “meta” package in R version 4.3.0 (http://www.r-project.org/index.html, accessed on 24 June 2023).

## 3. Results

### 3.1. Paper Selection Process

**Figure 1:**
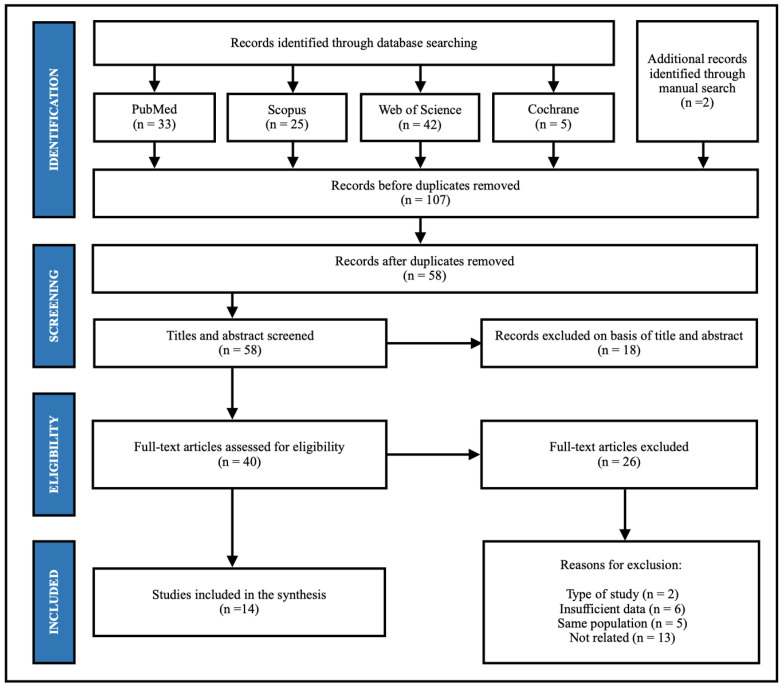
Flowchart of the study selection process.

A last search of all databases was conducted in May 2023. A total of 105 articles were retrieved through database searching, and two were retrieved through manual search. After removing duplicates (*n* = 56), 51 articles were screened using title-abstract-keyword reading, leaving 35 reports eligible (substantial agreement, κ = 0.95). After full-text reading and a subsequent search for relevant citations, 14 studies were included in this systematic review [8,10,11,12,13,14,15,19,32,33,34,35,36,37] (100% of agreement among the reviewers) (Figure 1). One current retrospective study from Wu included [19], and other fives studies from the same team (Shanghai Jiaotong University) were excluded due to the same population were collected in Wu’s publication [19]. Three studies investigated the guided approach for placing ZI but did not report placement deviation. Detailed reasons for excluding 20 articles are summarized in the additional file (Supplementary Materials Table S2).

### 3.2. Characteristics of the Included Studies

The main characteristics of the included studies are described in Table 1 according to guidance approach, system, number of examinations, ZI characteristics, and conclusions. A total of 14 studies [8,10,11,12,13,14,15,19,32,33,34,35,36,37], including one RCT [32], two retrospective studies [15,19], five prospective studies [12,13,33,34,37], six cadaver studies with one comparative analysis [8,10,11,14,35,36], were retrieved from the search. Those reported ZI planned/placed accuracy in 144 patients (77 male and 67 female) and 35 cadavers with total of 511 ZIs (Nobel Biocare: 274, Southern Implant: 42, SIN Implant: 16, non-mentioned: 179); six studies used 35 cadavers with 118 ZIs. Eight studies included 144 patients with 413 ZIs. Of 14 included studies, three studies utilized the dynamic navigation guidance for ZI placement in 102 patients; nine studies utilized the static surgical guide in 38 patients and 25 cadavers, one study placed free-hand ZI in four patients and one study compared surgical guide and free-hand approach in ten cadavers.

Three surgical protocols (classic, quad, and maxillary defects) were used depending on the degrees of alveolar atrophy and indications. The classic approach (placing one ZI on each side) was reported in eight studies comprising 67 patients and six cadavers. The quad zygoma approach (placing two ZIs on each side) was reported in eight studies, including 56 patients and 24 cadavers with 320 ZIs. In four studies, 21 patients and five cadavers with a unilateral or bilateral defects of maxilla received 70 ZIs. In ten patients and three cadavers, a flapless surgery via classic approach with navigation (10) and surgical guide (3) was performed.

In the navigation group [19,32,33], 57 patients were treated via the classic approach, 35 patients via the quad zygoma approach, and in ten patients ZIs were placed in cases of deficient maxilla. In the static guide group [8,10,11,12,13,14,15,34,35,36], nine patients and six cadavers were treated via the classic approach, 18 patients and 24 patients via the quad zygoma approach, and in 11 patients and five cadavers, ZIs were used for maxillary defect cases. In the freehand group [10,37], one patient was treated with the classic approach, and three patients and ten cadavers received a quad zygoma approach. In the three studies of dynamic navigation, the following surgical navigational systems were used: Vector Vision 2 (BrainLAB, AG, München, Germany), Navident (ClaroNav, Toronto, ON, Canada) VectorVision, Dcarer Implant Dynamic Navigation (DCARER Medical Technology, Suzhou, China).

The design of surgical guides could be divided into two groups: a conventional surgical guide and a modified guide. Four studies used the conventional surgical guide in which the sleeve was designed in a regular length (4–6 mm) for the implant drilling procedure [8,34,35,36]. This guide was supported by the bone in three studies and supported by the mucosal tissue in one study. Six studies used a modified surgical guide with double or extra-long sleeves designs [10,11,12,13,14,15]. The modified surgical guides were larger than conventional ones and were fixed with multiple screws to the bone. Here, two sleeves were used; one for implant site preparation at the alveolar processus level and one at the inferior zygomatic level.

Regarding the material used for fabricating the guides, four studies utilized 3D printed titanium material (EZgoma Guide, Noris Medical Ltd., Nesher, Israel and DePuy/Synthes, Materialise, Leuven, Belgium) in edentulous maxilla patients, and two studies used a 3D printed resin material with an extra-length metal sleeve in patients with a deficient maxilla (3-Matic Medical, Materialise, Leuven, Belgium) for producing the surgical guide.

**Table 1:** The characteristics of the included studies.

Author, Years	Study Type/Design	Number of Patient	Male/Female	Mean Age	Indication	Number of ZI	Implant Brand	Guide (System)	Prosthetically Driven Planning	Conclusion
Wu, 2022 [19]	Clinical Retrospective study	71	38/33	46.8	Severe atrophic edentulous maxilla/defected maxilla	221	Nobel Biocare	d-CAIS (BrainLAB, AG, Germany)	Yes	The navigation is an accurate and reliable surgical approach for ZI surgery, and it allows clinicians to accurately transfer preoperative planning to patients during surgery.
Bhalerao, 2023 [32]	RCT	20	16/4	57.2	Severe atrophic edentulous maxilla	20	NM	dCAIS (Navident, ClaroNav, Toronto, ON, Canada)	NM	Adequate training on the use of dynamic navigation is mandatory before its use in clinical cases.
Guo, 2023 [33]	Clinical Retrospective study	11	6/5	56	Severe atrophic edentulous maxilla	21	Nobel Biocare	dCAIS (Dcarer, DHC-DI242, China)	No	The actual positions of placed ZIs were slightly deviated from the ideal due to navigation errors.
Rinaldi, 2019 [34]	Clinical Prospective study	4	2/2	58.5	NM	10	Nobel Biocare	s-CAIS/(RealGUIDE 5.0)	No	Preparation of the sinus fenestration using the surgical guide.
Schiroli, 2016 [35]	Cadaver study	3	/	/	/	6	Nobel Biocare, Southern Implant	s-CAIS (SURGIGUIDE, Materialise Dental NV, Leuven, Belgium)	NM	Computer-guided flapless zygomatic implant surgery remains challenging.
Chrcanovic, 2010 [8]	Cadaver study	4	/	/	/	16	SIN Implant	s-CAIS (Peclab Ltd., Belo Horizonte, Brazil)	NM	The use of the zygomatic implant, in the context of this protocol, should probably be reevaluated because some large deviations were noted.
Steenberghe, 2003 [36]	Cadaver study	3	/	/	/	6	Nobel Biocare	s-CAIS (SurgiGuideA, Materialise, Leuven, Belgium)	NM	Zygoma drilling guides seem to offer an accurate tool to achieve a successful and reliable treatment outcome in the majority of cases.
Gallo, 2023 [15]	Clinical Retrospective study	19	8/11	61	Severe atrophic edentulous maxilla	59	NM	modified s-CAIS (EZgoma Guide, Noris Medical Ltd., Nesher, Israel)	Yes	Fully guided surgery showed good accuracy for ZI placement and it should be considered in the decision-making process.
Vosselman, 2022 [13]	Clinical Prospective study	10	3/7	66.3	Defected maxilla (Brown IIb)	28	Southern Implant	modified s-CAIS (3-Matic Medical, Materialise, Leuven, Belgium)	Yes	A fully digitalized workflow for guided resection and ZI placement is feasible.
Bolzoni, 2023 [12]	Clinical Prospective study	5	1/4	62.2	Severe atrophic edentulous maxilla	20	NM	modified s-CAIS (EZgomaGuide)	Yes	Guided ZI rehabilitation may represent a reliable, efficient, rapid, ergonomic, and safe surgical protocol; however, further investigations are needed.
Grecchi E, 2021 [10]	Cadaver study	10 *	/	/	/	20	NM	modified s-CAIS (EZgoma Guide, Noris Medical Ltd., Nesher, Israel)	NM	Guided surgery system exhibited a higher accuracy for all the investigated variables, when compared to the free-hand technique.
Grecchi F, 2021 [11]	Cadaver study	10	/	/	/	40	NM	modified s-CAIS (EZgomaGuide)	NM	In terms of accuracy and with respect to the planning, the procedure is feasible with successful results even if performed by unexperienced surgeons.
Vosselman, 2021 [14]	Cadaver study	5	/	/	/	10	Southern Implant	modified s-CAIS (3Matic Medical, Materialise, Leuven, Belgium)	Yes	ZIs should be placed accurately in the planned positions using the novel designed patient specific drilling and placement guides.
Gao, 2021 [37]	Clinical Prospective study	4	2/2	48.75	Severe atrophic edentulous maxilla	14	Nobel Biocare	FH (Planmeca Romexis^®^ 3D)	No	Virtual surgical planning is a useful tool helps the clinician determine the number and the length of ZIs as well as its proper position, surgical experience is still mandatory.
Grecchi E, 2021 [10]	Cadaver study	10 *	/	/	/	20	NM	FH (EZplan, NORIS medical, Israel)	NM	Guided surgery system exhibited a higher accuracy for all the investigated variables, when compared to the free-hand technique.

* share the sample size; dynamic computer-aided implant surgery (d-CAIS); static computer-aided implant surgery (s-CAIS); free-hand (FH); not mentioned (NM).

### 3.3. Risk of Bias

**Figure 2:**
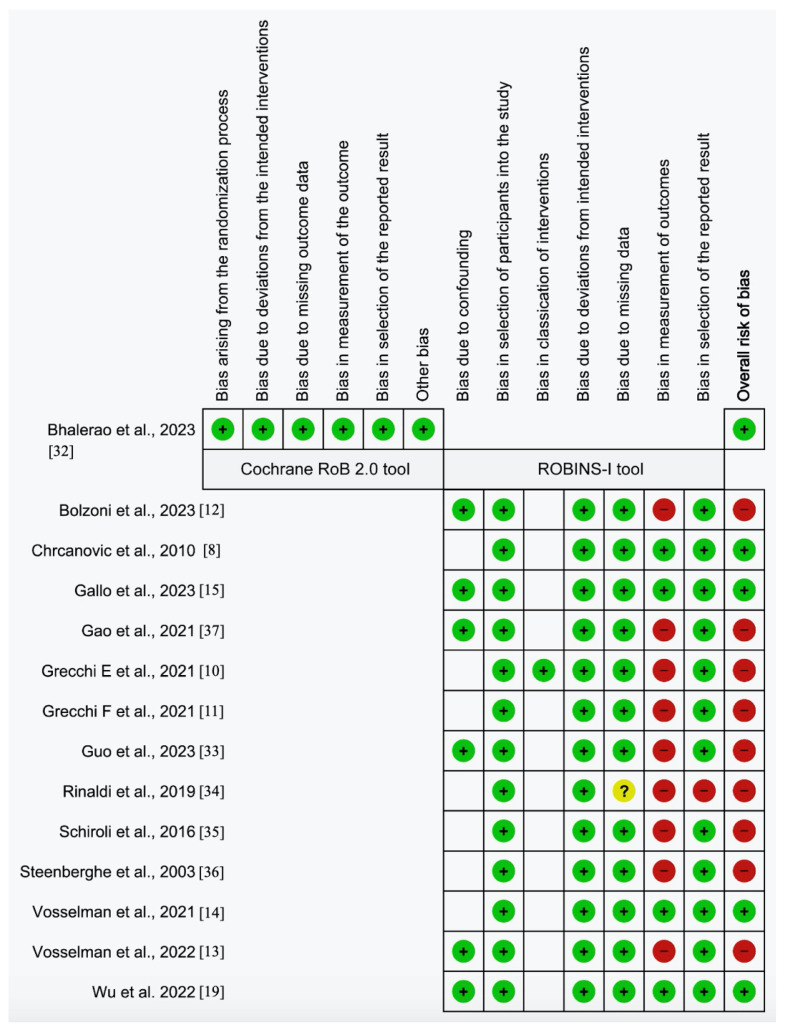
Results of risk of bias [8,10,11,12,13,14,15,19,32,33,34,35,36,37].

As the primary outcomes were measured using the same scale, the bias was summarized at the study level (Figure 2). Only one study [32] was evaluated using the Cochrane RoB 2.0 tool, which showed no serious concerns regarding its items, resulting in a low overall risk of bias. Regarding the thirteen included studies evaluated using the ROBINS-I tool, there were serious concerns regarding bias in measuring outcomes. Most studies did not report who conducted the accuracy assessment [10,11,12,13,33,34,35,36,37] or did not provide transparent reporting [19], potentially influencing their results. Additionally, the Rinaldi study should have reported a statistical analysis plan [34], raising additional concerns about bias in selecting reported results and potential issues with missing data. Finally, confirming essential information from Wu’s study helped clarify the risk of bias [19]. Regarding the secondary outcomes, there were no concerns regarding bias, as they were evaluated using a clinical, binary, and straightforward method, which reduced the chance of systematic error in their assessment.

### 3.4. Accuracy of ZI Placement

**Figure 3:**
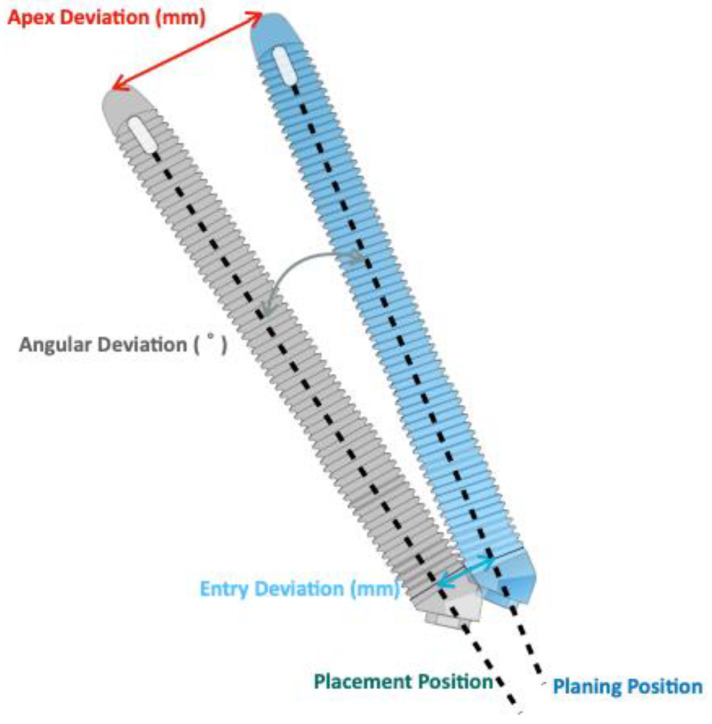
Illustration of the parameters of 3D deviation.

The deviation outcomes between the planned and placed positions were evaluated and described in two ways: two-dimensional (2D) and three-dimensional (3D) (Figure 3). Out of the 14 included studies, 12 studies analyzed the deviation outcomes using 3D measurements [10,11,12,13,14,19,32,33,34,35,36,37]. In contrast, two studies used 2D measurements [8,15]. The deviations reported in each study are summarized in Table 2. For the 2D measurements, Gallo et al. measured the linear differences at the entry and apex of the implant in the X-axis, Y-axis, and Z-axis directions, respectively [15]. The angular deviation between the planned/placed ZIs was determined in terms of yaw, pitch, and roll of the long axis of each implant. On the other hand, Chrcanovic et al. only measured the angular deviation between the planned/placed ZIs in the anterior-posterior view and caudal-cranial view of each implant [8].

**Figure 4:**
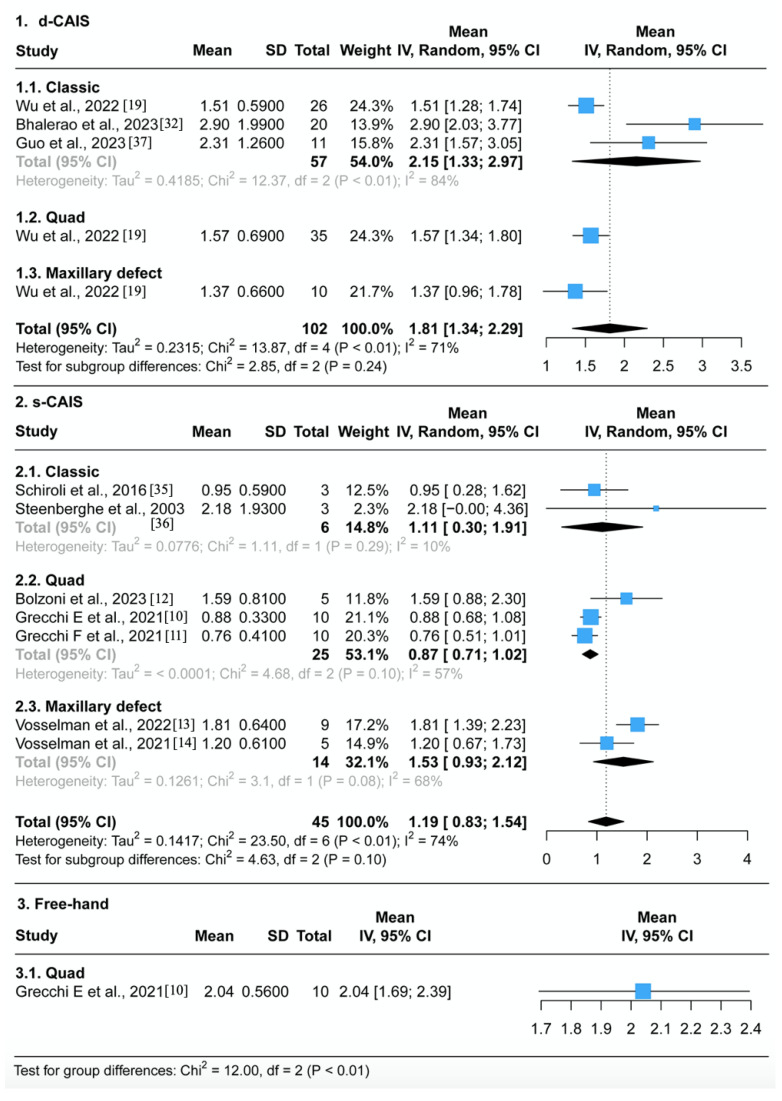
Forest plot representing the pooled mean 3D entry deviation grouped by different ZI surgical protocol in (1) the navigation group, (2) the guide group, (3) and the free-hand group [10,11,12,13,14,19,19,32,35,36,37].

**Figure 5:**
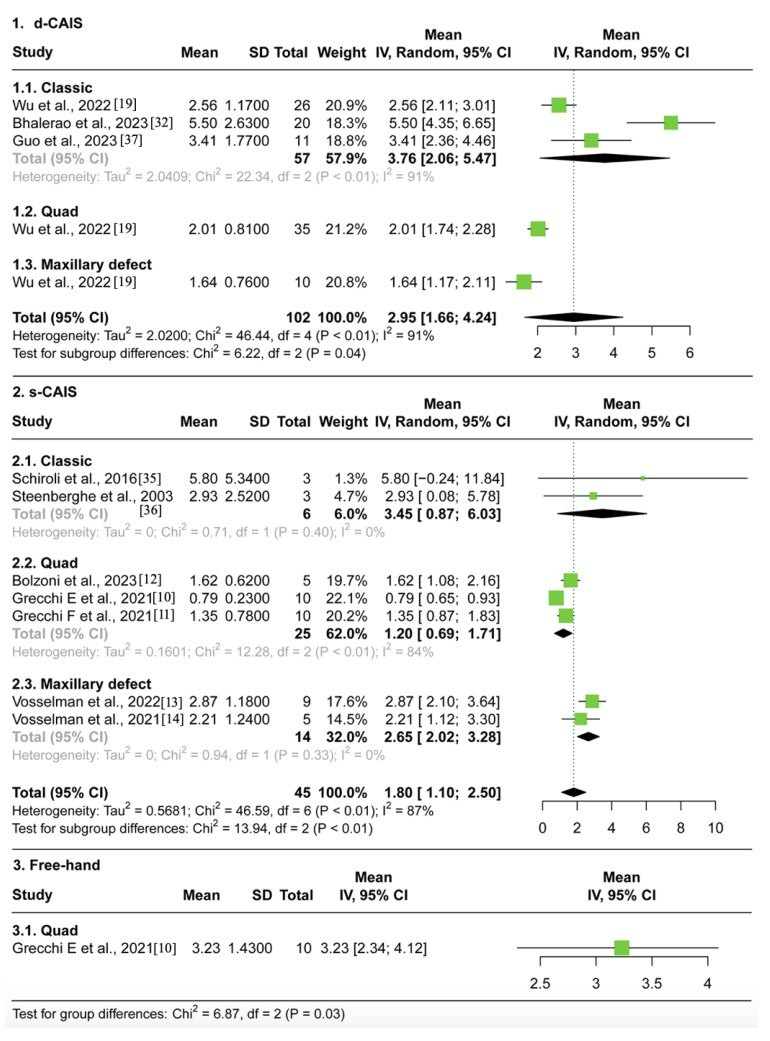
Forest plot representing the pooled mean 3D apex deviation grouped by different ZI surgical protocol in (1) the navigation group, (2) the guide group, (3) and the free-hand group [10,11,12,13,14,19,19,32,35,36,37].

**Figure 6:**
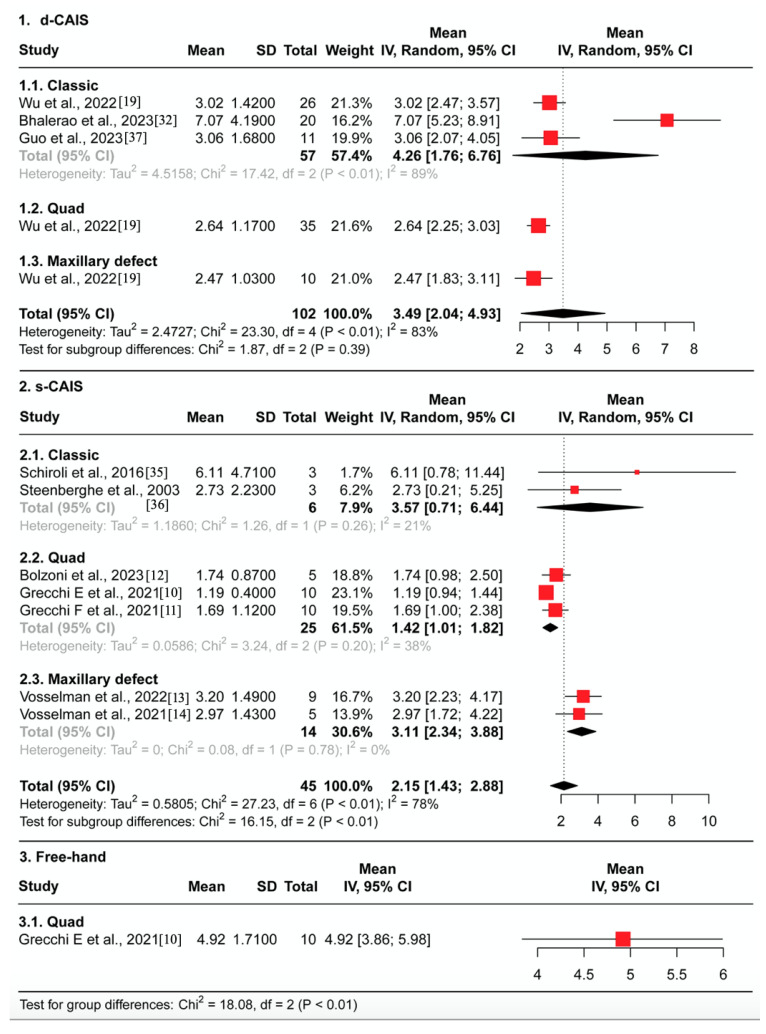
Forest plot representing the pooled mean angular deviation grouped by different ZI surgical protocol in (1) the navigation group, (2) the guide group, (3) and the free-hand group [10,11,12,13,14,19,19,32,35,36,37].

Regarding measurement methodology, all 14 studies used post-operative CT/CBCT scanning to merge with pre-operative planning images and superimpose the planned and placed outcomes using different software tools. The two studies that used 2D measurements were excluded from the subgroup analysis [8,15], and two studies with diverse surgical approaches without clarifying deviations were excluded from the meta-analysis [34,37]. The final selection of 10 studies was performed in qualitative and quantitative synthesis (Figure 4, Figure 5 and Figure 6).

**Table 2:** The deviations reported in included study.

Author, Years	Appraoch (*n*)	Mean Length	Entry Point (Range)	Exit Point (Range)	Angular (Range)	Accuracy Analysis (Software)
Dynamic computer-aided implant surgery						
Wu, 2022 [19]	Classic (26)	NM	1.51 ± 0.59 (0.40–3.15)	2.56 ± 1.17 (0.70–5.85)	3.02 ± 1.42 (0.75–5.60)	CBCT (I-plan, BrainLAB, AG, Germany)
	Quad (35)		1.57 ± 0.69 (0.15-3.7)	2.01 ± 0.81 (0.81)	2.64 ± 1.17 (0.45–5.75)	
	Defect maxilla (10)		1.37 ± 0.66 (0.45–3.1)	1.64 ± 0.76 (0.4–3.1)	2.47 ± 1.03 (0.15–4.45)	
Bhalerao, 2023 [32]	Classic-flapless (10)	NM	2.03 ± 1.96 (NM)	4.43 ± 2.07 (NM)	5.25 ± 3.32 (NM)	CBCT (EvaluNav, ClaroNav, Toronto, ON, Canada)
	Classic (10)		3.77 ± 1.69 (NM)	6.57 ± 2.79 (NM)	8.89 ± 4.33 (NM)	
Guo, 2023 [33]	Classic (10) Unilateral (1)	44.64	2.31 ± 1.26 (NM)	3.41 ± 1.77 (NM)	3.06 ± 1.68 (NM)	CBCT (Mimics Medical, materialize dental, Leuven, Belgium)
Static computer-aided implant surgery						
Rinaldi, 2019 [34]	Classic (3) Quad (1)	NM	3.55 (2.66–4.37)	2.11 (0.51–4.21)	4.55 (1.16–8.45)	CT (Mimics Medical, materialize dental, Leuven, Belgium)
Schiroli, 2016 [35]	Classic-flapless (3)	47.08	0.95 ± 0.59 (0.2–1.7)	5.8 ± 5.34 (0.9–15.5)	6.11 ± 4.71 (1.3–14.2)	CT (Mimics Medical, materialize dental, Leuven, Belgium)
Chrcanovic, 2010 [9] ^#^	Quad (4)	NM	/	/	anterior-posterior 11.20 ± 9.75 (0.35–21.20) caudal-cranial 11.20 ± 9.75 (0.76–37.60)	CT (SkillCrest, Tucson, Ariz)
Steenberghe, 2003 [36]	Classic (3)	45	2.18 ± 1.93 (0.7–6.0)	2.93 ± 2.52 (0.8–7.9)	2.73 ± 2.23 (0.61–6.93)	CT (NM)
Gallo, 2023 [15] ^#^	6 Classic (6) 12 Quad (12) Unilateral (1)	NM	NM (0.1–0.31)	NM (0.49–4.62)	NM (0.02–1.54)	CBCT (3DSlicer, version 4.13.0)
Vosselman, 2022 [13]	Defected maxilla (9)	46.83	1.81 ± 0.64 (0.43–3.24)	2.87 ± 1.18 (1.11–4.72)	3.20 ± 1.49 (0.34–6.13)	CBCT (NM)
Bolzoni, 2023 [12]	Quad (5)	45	1.59 ± 0.81 (0.54–3.23)	1.62 ± 0.62 (0.93–2.96)	1.74 ± 0.87 (0.71–4.25)	CT (Mimics Medical, materialize dental, Leuven, Belgium)
Grecchi E, 2021 [10]	Quad (10 *)	NM	0.88 ± 0.33 (NM)	0.79 ± 0.23 (NM)	1.19 ± 0.40 (NM)	CT (Mimics Medical, materialize dental, Leuven, Belgium)
Grecchi F, 2021 [11]	Quad (10)	NM	0.76 ± 0.41 (NM)	1.35 ± 0.78 (NM)	1.69 ± 1.12 (NM)	CBCT (Mimics Medical, materialize dental, Leuven, Belgium)
Vosselman, 2021 [14]	Defected maxilla (5)	NM	1.20 ± 0.61 (0.4–2.1)	2.21 ± 1.24 (0.7–4.1)	2.97 ± 1.43 (1.0–5.5)	CBCT (NM)
Free-hand						
Gao, 2021 [37]	Classic (1) Quad (3)	44.64	6.11 ± 4.28 (NM)	4.98 ± 2.66 (NM)	8.35 ± 5.30 (NM)	CT (Dolphin Imaging 11.95 Premium)
Grecchi E, 2021 [10]	Quad (10)	NM	2.04 ± 0.56 (NM)	3.23 ± 1.43 (NM)	4.92 ± 1.71 (NM)	CT (Mimics Medical, materialize dental, Leuven, Belgium)

* share the sample size; ^#^ analyzed the deviation outcomes in *n* 2D measurement.

#### 3.4.1. Entry Deviation (Figure 4)

- In the d-CAIS group, the average entry deviation was 1.81 mm, with solid evidence of heterogeneity (95% CI 1.34–2.29, I^2^ = 71%, *p* < 0.01), across 102 patients from three studies. No significant differences were observed between the classic, quad, and maxillary defected groups (*p* > 0.1).
- In the s-CAIS group, the average entry deviation was found to be 1.19 mm, with solid evidence of heterogeneity (95% CI 0.83–1.54, I^2^ = 74%, *p* < 0.01), across a total of 14 patients from two studies and 31 cadavers from five studies. There were slightly significant differences observed between the classic, quad, and maxillary defected groups (*p* = 0.1).
- In the free-hand group, the average entry deviation was 2.04 mm (95% CI 1.69–2.39) across ten patients from one study.
- There was strong evidence of differences in the average entry deviation between the navigation, surgical guide, and free-hand groups (*p* < 0.01). ZI placement assisted by d-CAIS was less accurate than s-CAIS but had less deviation compared to the free-hand approach.
- Sensitivity analysis reveals no impact of risk of bias on effect estimates when comparing low risk of bias and high risk of bias studies for d-CAIS (Chi^2^ = 1.65, df = 1, *p* = 0.20). However, for s-CAIS, there is a difference between low risk (1.81 mm, 95%CI 1.39–2.23) and high risk of bias studies (0.94 mm, 95% CI 0.74–1.14) (Chi^2^ = 13.38, df = 1, *p* < 0.01), but not for type of study (cadaveric vs. clinical) (Chi^2^ = 0.09, df = 1, *p* = 0.77).

#### 3.4.2. Apex Deviation (Figure 5)

- In the d-CAIS group, the average apex deviation was 2.95 mm, with strong evidence of heterogeneity (95% CI 1.66–4.24, I^2^ = 91%, *p* < 0.01), across 102 patients from three studies. Significant differences were observed between the classic, quad, and maxillary defects groups (*p* < 0.1).
- In the s-CAIS group, the average apex deviation was found to be 1.80 mm, with strong evidence of heterogeneity (95% CI 1.10–2.50, I^2^ = 87%, *p* < 0.01), across a total of 14 patients from two studies and 31 cadavers from five studies. Significant differences were observed between the classic, quad, and maxillary defects groups (*p* < 0.01).
- In the free-hand group, the average apex deviation was found to be 3.23 mm (95% CI 2.34–4.12) across a total of 10 patients from one study.
- Significant differences were in the average apex deviation between the navigation, surgical guide, and free-hand groups (*p* = 0.03). ZI placement assisted by d-CAIS was less accurate than s-CAIS but had less deviation compared to the free-hand approach.
- Sensitivity analysis reveals no impact of risk of bias on effect estimates when comparing low risk of bias and high risk of bias studies for d-CAIS (Chi^2^ = 0.31, df = 1, *p* = 0.58). For s-CAIS, there is a difference in effect estimates between low risk (2.87 mm, 95% CI 2.10–3.64) and high risk of bias studies (1.46 mm, 95% CI 0.90–2.01) (Chi^2^ = 8.48, df = 1, *p* < 0.01), but not for type of study (cadaveric vs. clinical) (Chi^2^ = 1.16, df = 1, *p* = 0.28).

#### 3.4.3. Angular Deviation (Figure 6)

- In the d-CAIS group, the average angular deviation was found to be 3.49 degrees, with strong evidence of heterogeneity (95% CI 2.04–4.93, I^2^ = 83%, *p* < 0.01), across a total of 102 patients from three studies. No significant differences were observed between the classic, quad, and maxillary defects groups (*p* > 0.1).
- In the s-CAIS group, the average angular deviation was found to be 2.15 degrees, with strong evidence of heterogeneity (95% CI 1.43–2.88, I^2^ = 78%, *p* < 0.01), across a total of 14 patients from two studies and 31 cadavers from five studies. Significant differences were observed between the classic, quad, and maxillary defects groups (*p* < 0.1).
- In the free-hand group, the average angular deviation was found to be 4.92 degrees (95% CI 3.86–5.98) in total of 9 patients from one study.
- There is strong evidence of differences in the average angular deviation between the navigation, surgical guide, and free-hand groups (*p* < 0.01). ZI placement assisted by d-CAIS was less accurate than s-CAIS but had less deviation compared to the free-hand approach.
- Sensitivity analysis reveals no impact of risk of bias on effect estimates when comparing low risk of bias and high risk of bias studies for d-CAIS (Chi^2^ = 0.28, df = 1, *p* = 0.60). Regarding s-CAIS, there is differences between the effect estimates of low risk (3.2 degrees, 95% CI 2.23–4.17) and high risk of bias studies (1.84 degrees, 95% CI 1.21–2.46) (Chi^2^ = 5.34, df = 1, *p* = 0.02) but not for type of study (cadaveric vs. clinical) (Chi^2^ = 1.69, df = 1, *p* = 0.19).

#### 3.4.4. Meta-Regression Analysis: Number of Fiducial Screws for Registration in Navigation Approach

All three studies in the d-CAIS group utilized invasive bone screws as fiducial markers to perform registration, with different numbers and configurations. A significant inverse correlation was observed between the number of fiducial screws and the planned/placed deviation in terms of entry, apex, and angular measurements (Table 3). A higher number of fiducial screws inserted for registration resulted in less final deviation in the navigation approach.

**Table 3:** Meta-regression models.

d-CAIS Group			
Deviation	Entry	Apex	Angular
Amount of screws for registration (less to more)	B = −0.27 [95% CI −0.42,−0.12], *p* = 0.0003, R^2^ = 100%	B = −0.62 [95% CI −0.91,−0.32], *p* < 0.0001, R^2^ = 88.8%	B = −0.59 [95% CI −1.14,−0.04], *p* = 0.036, R^2^ = 53.9%
**s-CAIS group**			
**Deviation**	**Entry**	**Apex**	**Angular**
Type of surgical guide (Conventional vs. Modified)	B = 0.04 [95% CI −0.99,−1.08], *p* = 0.94, R^2^ = 0%	B = −1.84 [95% CI −4.76,1.09], *p* = 0.21, R^2^ = 3.2%	B = −1.43 [95% CI −4.13,1.27], *p* = 0.30, R^2^ = 0%

#### 3.4.5. Meta-Regression Analysis: Conventional and Modified Surgical Guided System

In the s-CAIS group, two studies utilized the conventional guide design and five studies utilized a modified guide with double sleeves technique for placing ZI. No significant difference was observed between the two designs of guides and planed/placed deviation in terms of entry, apex, and angular measurements (Table 3).

### 3.5. Survival Rate and Complication

Four clinical studies reported the ZI survival rate ranged 98.64% to 100% and three studies reported navigation/surgical-related complications were summarized in Table 4.

**Table 4:** Survival and complication report.

	Author, Years	Survival (Follow-Up)	Surgical/Navigation-Related Complication
1	Wu, 2022 [19]	98.64% (24.11 M)	28 device-related negative events, and one resulted in 2 ZIs failures due to implant malposition
3	Guo, 2023 [33]	100% (6 M)	NM
12	Chrcanovic, 2010 [8]	/	One implant emerged inside the orbital cavity One implant emerged in the infratemporal fossa
4	Gallo, 2023 [15]	100% (6 M)	NM
6	Bolzoni, 2023 [12]	100% (15.9 M)	Fracture of the anterior wall of the maxillary sinus

M: month; NM: not mentioned.

### 3.6. Publication Bias

**Figure 7:**
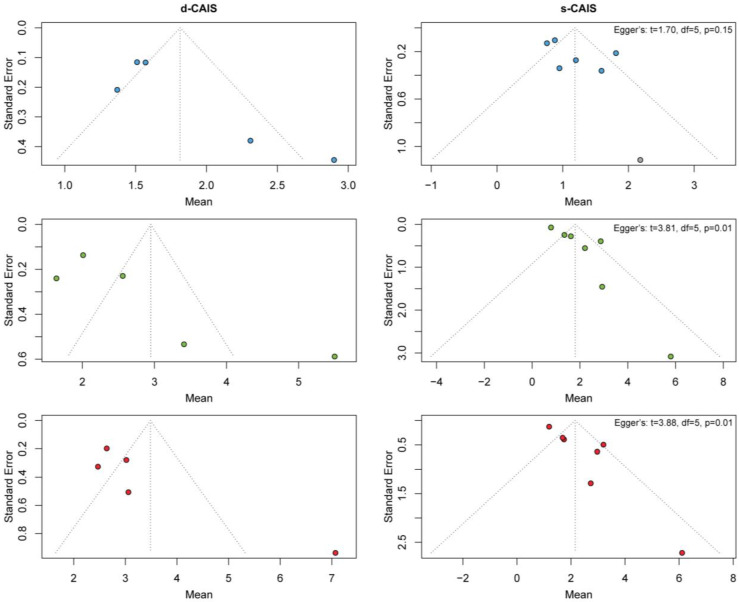
The funnel plot was used for the visual inspection of asymmetry in the navigation and surgical guide groups for entry, apex and angular deviation. Entry deviation in blue. Apex deviation in green. Angular deviation in red.

The visual inspection of funnel plot asymmetry shows a tendency for asymmetry for navigation and surgical guide in each of the three primary outcomes. However, this is only confirmed with Egger’s test for apex and angular deviation (*p* < 0.1) (Figure 7). Publication bias cannot be evaluated in the free-hand method, as only one study was included.

## 4. Discussion

The present systematic review aimed to evaluate the existing literature on the planned and placed deviations in ZI placement with surgical navigation system guidance, and compare it to static surgical guide and free-hand approaches in severely atrophic edentulous maxilla and/or maxillary defects. The results demonstrated that utilizing d-CAIS and modified s-CAIS for ZI placement yielded clinically acceptable outcomes in terms of average entry, apex, and angular deviations. The utilization of d-CAIS and s-CAIS resulted in an average entry and apex deviation of less than 3 mm. The accuracy achieved through the free-hand approach primarily relied on the surgeon’s experience and learning curve. Strong evidence for significant differences in the average entry and angular deviation between the navigation, surgical guide, and free-hand groups had been found. The results showed that ZI placement assisted by d-CAIS was less accurate than s-CAIS but had less error compared to the free-hand approach. However, the meta-analysis encountered issues of inconsistency (high heterogeneity) and imprecision (wide confidence intervals) due to the diverse clinical diversity in study designs and surgical protocols, as well as differences in the effect estimates of s-CAIS between categories of risk of bias (low vs. high risk of bias studies). Several limitations were identified in various aspects.

The deviation of planned/placed implant placement is typically described using the mean value. The safe zone for planning conventional implant surgery is usually considered to be within 2–3 mm, which is an acceptable error value to avoid injury to critical anatomical structures. However, the maximum value of deviation for each guide approach should be addressed during the surgery when the critical anatomical structures are nearby the surgical field, even if it is a rare occurrence. Schiroli et al. used a conventional guide with a mucosa-supported template for placing ZI [35]. In this cadaver study, the maximum deviation at the apex was 15.5 mm with a flap-less approach. Although no complications were reported in this study, the results suggested a high risk of penetrating the orbital or infra-temporal fossa with this technique. Similar results were also registered with a bone-supported surgical guide. Chrcanovic and Steenberghe showed angular and apex deviations up to 37.6 degrees and 7.9 mm, respectively [8,36]. In Chrcanovic’s cadaver study, a massive angular deviation resulted in one ZI emerging inside the orbital cavity and another in the infra-temporal fossa [8]. These findings highlight that with the different levels of the atrophic maxilla, the fixation of surgical guides is challenged by uncorrected positioning and instability, primarily due to insufficient tissue support underneath.

The modified surgical guide with a double sleeves design has successfully addressed the issue of maintaining the stability of a nearly 100 mm surgical drill and located the entry point in the zygoma bone with two titanium sleeves. However, the author identified two limitations with the double sleeves guide design. Firstly, due to the design of the surgical guide, the trajectory of ZI insertion is limited to the extra-sinus pathway [10,11,12,15], and intra-sinus and para-maxillary sinus pathways cannot be performed using this concept. A cross-sectional study by Aparicio classified 200 patients’ radiological images into five groups (ZAGA0-ZAGA4) based on individual alveolar atrophy level and the degree of maxillary lateral wall curvature [38]. The distribution of patients across the groups was as follows: intra-sinus (ZAGA0) 15%, para-maxillary sinus (ZAGA1 and 2) 49% and 20.5%, and extra-sinus (ZAGA3 and 4) 9% and 6.5%, respectively. Davo’s clinical study described the distribution of 182 ZIs placement as 5% intra-sinus, 52% on the sinus wall, and 42% via an extra-sinus pathway [39]. Therefore, using the double sleeves guide for ZI placement in intra/para-sinus pathways might be limited due to individual anatomical differences.

Secondly, the trajectory of ZI is fixed throughout the entire operation when utilized s-CAIS. However, CBCT scans cannot provide 100% accurate information on factors such as the patient’s mouth opening, local infection, and bone healing in the residual alveolar ridge [40]. Changes in planning may be necessary when encountering these situations. In contrast, navigation tracking techniques offer the flexibility to adapt pre-operative planning to the surgical site, enabling a better anchorage position for ZI placement [16]. For changing an entry or apex point, using the navigation probe to touch the newly designed entry point or setting up the monitor could be newly defined [18].

In one eligible study conducted by Guo, bone-implant contact (BIC) was investigated in 11 patients who underwent the classic approach with navigation guidance in the zygoma bone area [33]. The radiological BIC measurements were obtained for 21 ZIs and showed an average of 15.17 ± 4.67 mm on the facial-temporal view. In Wang’s study, 52 ZIs were placed using navigation guidance. All ZIs achieved osseointegration, and an immediate loading protocol was performed, resulting in an overall survival rate of 100% [41]. The average BIC of the ZIs in this study was 14.5 mm, with 11 cases using the quad zygoma approach and 4 cases using the classic approach. For the quad zygoma approach, placing 2 ZIs with optimal distribution in zygomatic bone is more challenging. When considering the expected deviation, it is crucial to ensure a minimum of 2 mm inter implant distance at the ZI apex which is necessary to ensure adequate osseointegration around implants. Additionally, it is imperative to maintain a safe distance of 2 mm from the orbital cavity and the infra-temporal fossa during the planning and surgical procedures.

As a crucial part of navigation surgery, registration is defined as the determination of the spatial relationships between the virtual coordinate system and the intra-operative patient coordinate system, and its precision is vital to the actual navigation surgery [42]. The use of the invasive bone anchorage screw is considered the gold standard with high accuracy for the registration procedure [43]. The present review highlights a crucial key fact: a significant correlation was observed between the number of fiducial screws for registration and the planned/placed deviation. Fan et al. evaluated the registration error in 9 combinations of fiducial screws in different distributions by inserting the screw in the anterior nasal spine, bilateral maxillary tuberosity, and midline palatine suture, with 4 to 10 screws [44]. The study showed that using at least five fiducial screws in the edentulous maxilla for registration achieves an acceptable value for ZI navigation surgery. However, in clinical practice, adding 2–3 additional screws was suggested to avoid individual imaging errors of metal artifacts and screw loosening during the operation. In Wu’s study, 12 fiducial screws in 10 patients were reported to have loosened and dropped out during the registration procedure [19]. Due to sufficient screws being inserted in its protocol, no significant difference was found in the entry, exit, and angle deviations between the stable and loosening groups.

A substantial body of evidence has supported the long-term success of ZI rehabilitation in the edentulous maxilla and maxillary deficiency using the free-hand approach [45,46]. However, only two eligible studies have followed pre-operative planning and performed the free-hand approach for placing ZIs with reporting accuracy in the present systematic review [10,37]. Both studies concluded that surgical experience is essential for surgeons planning and performing ZI surgery. In Gao’s analysis, ten ZIs were placed in four patients, resulting in entry deviation, apex deviation, and angular deviation of 6.11 mm, 4.98 mm, and 8.35 degrees, respectively [37]. In a cadaver study, a comparison between the modified surgical guide and the free-hand approach demonstrated a significant difference, which with the guide exhibited higher accuracy than the free-hand approach [10]. Another study compared the accuracy between the free-hand and navigation approach in 15 polyurethane models [47]. The results showed that the free-hand approach was more accurate than navigation in apical deviations. However, it is essential to note that the operations were performed in standard models with direct visualization of the entire surgical field, which may not fully reflect the clinical challenges faced when performing ZI treatment.

Utilizing high-quality CBCT or CT data for planning, producing, and executing surgical guides or navigation operations has shown similar results in surgery accuracy [48,49]. To analyze the planned/placed error, all eligible studies performed at least one post-operative CBCT or CT scan to merge with the pre-operative planning. The software enables the automatic imposition of pre- and post-implant positions to evaluate the outcomes. However, it is essential to note that radiation dose needs to be carefully considered and controlled for patients treated with digital approaches. Particularly in the case of navigation approaches, additional image scanning is required for fiducial screw insertion to perform real-time guidance registration [16]. Radiation exposure should be considered, as demonstrated in a systematic review, due to the necessity of 3D imaging for CAIS [50]. Navigation-assisted implant placement should be considered more carefully, especially in complex cases. An in vitro study compared two navigation systems, innovative registration, and point-to-point registration, to evaluate the accuracy of ZI placement [51]. The results showed no significant deviation difference between the two groups, but innovative registration methods allowed for minimally invasive surgery by avoiding the insertion of fiducial screws and additional CT scans. Future studies should validate the use of intra-oral scanning for error measurement purposes to reduce radiation exposure and artifacts in the images.

The present study demonstrated that the mean deviation of entry, apex, and angular measurements was lower in the s-CAIS group compared to the d-CAIS group, as reported in the pooled descriptive statistics. This finding can be attributed to the limitations of the existing literature, which prevented the inclusion of homogenized studies. Within the d-CAIS group, all the studies were clinical research involving 102 patients. On the other hand, the s-CAIS group consisted of only two clinical studies with 14 patients, while the remaining five were cadaver studies with 31 samples. Direct statistical comparisons between different approaches were impossible due to the inclusion of only one comparative study and the differences in study types, surgical techniques, and lack of procedure description among the included studies. Future studies should focus on RCTs, comparing the feasibility of different guide approaches in large sample sizes and evaluating other guide systems regarding long-term outcomes and learning curves.

## 5. Conclusions

With the assistance of the surgical navigation system, ZI placement can achieve acceptable clinical accuracy through real-time visualization and tracking techniques. Registration is a crucial factor that can influence treatment outcomes, and a correlation has been observed between the number of invasive screws used as fiducial markers and the planned/placed error. The modified surgical guide for ZI surgery also appears to yield clinically acceptable outcomes, although future studies are needed to validate its feasibility and reliability in clinical research. The accuracy and safety achieved through the free-hand approach mainly depend on the surgeon’s experience and skills. The maximal deviation was observed primarily on the conventional guide; however, it should be considered in each approach. The factors contributing to maximal deviation should be investigated in more studies with proper protocols to address and minimize it. Furthermore, using a flapless approach in ZI placement should be cautiously approached due to the potential for deviation and associated complications.

## Data Availability

The data sets used and/or analyzed during the current study are available from the corresponding author on reasonable request.

## Supplementary Materials

The following supporting information can be downloaded at: https://www.mdpi.com/article/10.3390/jcm12165418/s1, Table S1: Search queries; Table S2: Characteristics of the excluded studies.
