# Supplementary material for: The Accuracy of Zygomatic Implant Placement Assisted by Dynamic Computer-Aided Surgery: A Systematic Review and Meta-Analysis
Source: J Clin Med. 2023 Aug 21;12(16):5418. doi: 10.3390/jcm12165418 (PMC10455221; doi:10.3390/jcm12165418)
Supplement: Supplementary file 1 [file jcm-12-05418-s001.zip › jcm-2496198-supplementary.pdf]

Supplementary File

Table S1. Search queries.

Databases and registries	Search algorithm	Results
PubMed	("surgery, computer-assisted"[MeSH Terms]) OR ("guided surgery")) OR ("navigation surgery")) OR ("navigation system")) OR ("navigation systems")) OR ("real-time system")) OR ("real-time navigation")) OR ("dynamic guided surgery")) OR ("dynamic computer aided")) OR ("dynamic computer assisted")) OR ("computer-aided surgery")) OR ("freehand")) AND ("zygomatic implant")) OR ("zygomatic implants")) OR ("zygoma implant")) OR ("zygoma implants")) OR ("zygomatic fixture")) AND ("accuracy")	33
Scopus	(TITLE-ABS-KEY ("computer-assisted surgery") OR TITLE-ABS-KEY ("guided surgery") OR TITLE-ABS-KEY ("navigation surgery") OR TITLE-ABS-KEY ("navigation system") OR TITLE-ABS-KEY ("navigation systems") OR TITLE-ABS-KEY ("real-time system") OR TITLE-ABS-KEY ("real-time navigation") OR TITLE-ABS-KEY ("dynamic guided surgery") OR TITLE-ABS-KEY ("dynamic computer aided") OR TITLE-ABS-KEY ("dynamic computer assisted") OR TITLE-ABS-KEY ("computer-aided surgery") OR TITLE-ABS-KEY ("freehand")) AND (TITLE-ABS-KEY ("zygomatic implant") OR TITLE-ABS-KEY ("zygomatic implants") OR TITLE-ABS-KEY ("zygoma implant") OR TITLE-ABS-KEY ("zygoma implants") OR TITLE-ABS-KEY ("zygomatic fixture")) AND (TITLE-ABS-KEY ("accuracy"))	25
Web of Science	((((((((((((((ALL=("computer-assisted surgery")) OR ALL=("guided surgery")) OR ALL=("navigation surgery")) OR ALL=("navigation system")) OR ALL=("navigation systems")) OR ALL=("real-time system")) OR ALL=("real-time navigation")) OR ALL=("dynamic guided surgery")) OR ALL=("dynamic computer aided")) OR ALL=("dynamic computer assisted")) OR ALL=("computer-aided surgery")) OR ALL=("freehand")) AND ALL=("zygomatic implant")) OR ALL=("zygomatic implants")) OR ALL=("zygoma implant")) OR ALL=("zygoma implants")) AND ALL=("accuracy")	42
Cochranre Library	("computer-assisted surgery" OR "guided surgery" OR "navigation surgery" OR "navigation system" OR "navigation systems" OR "real-time system" OR "real-time navigation" OR "dynamic guided surgery" OR "dynamic computer aided" OR "dynamic computer assisted" OR "computer-aided surgery" OR "freehand") AND ("zygomatic implant" OR "zygomatic implants" OR "zygoma implant" OR "zygoma implants" OR "zygomatic fixture") AND ("accuracy")	5

Table S2. Characteristics of the excluded studies

**Reference**	**Excluded by wrong**

	P	I	C	O	T
Cao et al. 2019 [1]	●	○	○	○	●
Rigo et al. 2021 [2]	●	○	○	○	●
Stefanelli et al. 2020 [3]	●	●	○	○	○
Stefanelli et al. 2020 [4]	●	●	○	○	○
Qin et al. 2019 [5]	○	●	○	●	○
Yildirim et al. 2017 [6]	●	○	●	○	○
Li et al. 2020 [7]	○	●	○	●	○
Pena 2008 [8]	○	●	○	●	○
Fitzwater et al. 2011 [9]	●	○	●	○	○
Hung et al. 2018 [10]	○	●	○	●	○
Stefanelli 2020 [11]	●	●	○	○	○
Sverzut et al. 2022 [12]	○	●	○	○	○
Rubio 2008 [13]	●	●	●	●	○
Qin et al. 2019 [14]	○	●	○	○	○
Fernández-Olarte et al. 2021 [15]	○	●	○	○	○
Clinical protocol 1 [16]	●	○	○	●	○
Clinical protocol 2 [17]	●	○	○	●	○
Clinical protocol 3 [18]	●	○	○	●	○
Pellegrino et al. 2020 [19]	○	○	○	●	○
Chow et al. 2006 [20]	○	○	○	●	○

Schiroli et al. 2011 [21]	○	○	○	●	○
Zhou et al. 2020 [22]*	○	○	○	●	○
Hung et al. 2017 [23]*	○	○	○	●	○
Tao et al. 2020 [24]*	○	○	○	●	○
Wang et. al 2022 [25]*	○	○	○	●	○
Shen et al. 2021[26] *	○	○	○	●	○
* Fives studies from the same team (Shanghai Jiaotong University) were excluded due to the date were collected in Wu's publication.					

1. Cao Z, Qin C, Fan S, Yu D, Wu Y, Qin J, Chen X. Pilot study of a surgical robot system for zygomatic implant placement. Med Eng Phys. 2020 Jan;75:72-78. doi: 10.1016/j.medengphy.2019.07.020. Epub 2019 Oct 31. PMID: 31677890.
2. Rigo L, Tollardo J, Giammarinaro E, Covani U, Caso G. Fully Guided Zygomatic Implant Surgery. J Craniofac Surg. 2021 Nov-Dec 01;32(8):2867-2872. doi: 10.1097/SCS.0000000000008005. PMID: 34320580.
3. Stefanelli LV, Mandelaris GA, Franchina A, Di Nardo D, Galli M, Pagliarulo M, Testarelli L, Di Carlo S, Gambarini G. Accuracy Evaluation of 14 Maxillary Full Arch Implant Treatments Performed with Da Vinci Bridge: A Case Series. Materials (Basel). 2020 Jun 22;13(12):2806. doi: 10.3390/ma13122806. PMID: 32580340; PMCID: PMC7344455.
4. Stefanelli LV, Graziani U, Pranno N, Di Carlo S, Mandelaris GA. Accuracy of Dynamic Navigation Surgery in the Placement of Pterygoid Implants. Int J Periodontics Restorative Dent. 2020 Nov/Dec;40(6):825-834. doi: 10.11607/prd.4605. PMID: 33151187.
5. Qin C, Cao Z, Fan S, Wu Y, Sun Y, Politis C, Wang C, Chen X. An oral and maxillofacial navigation system for implant placement with automatic identification of fiducial points. Int J Comput Assist Radiol Surg. 2019 Feb;14(2):281-289. doi: 10.1007/s11548-018-1870-z. Epub 2018 Oct 13. PMID: 30317436.
6. Yildirim G, Kocaelli HA. Assessment of the content and quality of YouTube videos related zygomatic implants: A content-quality analysis. Clin Implant Dent Relat Res. 2023 Jun;25(3):605-612. doi: 10.1111/cid.13194. Epub 2023 Feb 20. PMID: 36808698.
7. Li C, Wang M, Deng H, Li S, Fang X, Liang Y, Ma X, Zhang Y, Li Y. Autonomous robotic surgery for zygomatic implant placement and immediately loaded implant-supported full-arch prosthesis: a preliminary research. Int J Implant Dent. 2023 May 19;9(1):12. doi: 10.1186/s40729-023-00474-2. PMID: 37204483; PMCID: PMC10199156.
8. Pena N, Campos PS, de Almeida SM, Bóscolo FN. Determination of the length of zygomatic implants through computed tomography: establishing a protocol. Dentomaxillofac Radiol. 2008 Dec;37(8):453-7. doi: 10.1259/dmfr/16676031. PMID: 19033430.
9. Fitzwater KL, Marcellin-Little DJ, Harrysson OL, Osborne JA, Poindexter EC. Evaluation of the effect of computed tomography scan protocols and freeform fabrication methods on bone biomodel accuracy. Am J Vet Res. 2011 Sep;72(9):1178-85. doi: 10.2460/ajvr.72.9.1178. PMID: 21879975.
10. Hung KF, Ai QY, Fan SC, Wang F, Huang W, Wu YQ. Measurement of the zygomatic region for the optimal placement of quad zygomatic implants. Clin Implant Dent Relat Res. 2017 Oct;19(5):841-848. doi: 10.1111/cid.12524. Epub 2017 Aug 1. PMID: 28766912.
11. Stefanelli LV, Pranno N, De Angelis F, La Rosa S, Polimeni A, Di Carlo S. Navigated Antral Bone Expansion (NABE): a prospective study on 35 patients with 4 months of follow-up post implant loading. BMC Oral Health. 2020 Oct 7;20(1):273. doi: 10.1186/s12903-020-01268-3. PMID: 33028292; PMCID: PMC7542702.
12. Sverzut TFV, Sverzut AT, Trivellato AE, Sverzut CE. Retrospective analysis of the predictability of using three-dimensional models for preoperative planning of the length of zygomatic implants. Oral

Maxillofac Surg. 2023 Mar;27(1):53-58. doi: 10.1007/s10006-022-01047-0. Epub 2022 Feb 15. PMID: 35166998.

13. Rubio Serrano M, Albalat Estela S, Peñarrocha Diago M, Peñarrocha Diago M. Software applied to oral implantology: update. Med Oral Patol Oral Cir Bucal. 2008 Oct 1;13(10):E661-5. PMID: 18830177.
14. Qin C, Ran X, Wu Y, Chen X. The development of non-contact user interface of a surgical navigation system based on multi-LSTM and a phantom experiment for zygomatic implant placement. Int J Comput Assist Radiol Surg. 2019 Dec;14(12):2147-2154. doi: 10.1007/s11548-019-02031-y. Epub 2019 Jul 12. PMID: 31300964.
15. Fernández-Olarte H, Gómez-Delgado A, Gutiérrez-Quintero JG, Rodríguez-Sáenz Á, Castro-Núñez J. The Morpho-Functional Three-Dimensional Analysis for Zygomatic Implants: A Clinical Tool With Surgical Implications. J Craniofac Surg. 2021 May 1;32(3):e254-e257. doi: 10.1097/SCS.0000000000006978. PMID: 32890146.
16. Comparision Between The Accuracy of Implants Placed in Weaken Upper Jaw Using Conventional Method and Computerised Method
17. Comparision of Effeciency Between the Traditional Method and Using a Computerised method in Placing Dental Implants
18. Navigation system contrast guide technology used to evaluate the accuracy of zygomatic implant placement: parallel, single-blind, randomized, controlled trial
19. Pellegrino G, Basile F, Relics D, Ferri A, Grande F, Tarsitano A, Marchetti C. Computer-Aided Rehabilitation Supported by Zygomatic Implants: A Cohort Study Comparing Atrophic with Oncologic Patients after Five Years of Follow-Up. J Clin Med. 2020 Oct 12;9(10):3254. doi: 10.3390/jcm9103254. PMID: 33053696; PMCID: PMC7600816.
20. Chow J, Hui E, Lee PK, Li W. Zygomatic implants--protocol for immediate occlusal loading: a preliminary report. J Oral Maxillofac Surg. 2006 May;64(5):804-11. doi: 10.1016/j.joms.2006.01.021. PMID: 16631489.
21. Schiroli G, Angiero F, Silvestrini-Biavati A, Benedicenti S. Zygomatic implant placement with flapless computer-guided surgery: a proposed clinical protocol. J Oral Maxillofac Surg. 2011 Dec;69(12):2979-89. doi: 10.1016/j.joms.2011.03.050. Epub 2011 Aug 11. PMID: 21835528.
22. Zhou W, Fan S, Wang F, Huang W, Jamjoom FZ, Wu Y. A novel extraoral registration method for a dynamic navigation system guiding zygomatic implant placement in patients with maxillectomy defects. Int J Oral Maxillofac Surg. 2021 Jan;50(1):116-120. doi: 10.1016/j.ijom.2020.03.018. Epub 2020 Jun 2. PMID: 32499080.
23. Hung KF, Wang F, Wang HW, Zhou WJ, Huang W, Wu YQ. Accuracy of a real-time surgical navigation system for the placement of quad zygomatic implants in the severe atrophic maxilla: A pilot clinical study. Clin Implant Dent Relat Res. 2017 Jun;19(3):458-465. doi: 10.1111/cid.12475. Epub 2017 Feb 20. PMID: 28219120.
24. Tao B, Shen Y, Sun Y, Huang W, Wang F, Wu Y. Comparative accuracy of cone-beam CT and conventional multislice computed tomography for real-time navigation in zygomatic implant surgery. Clin Implant Dent Relat Res. 2020 Dec;22(6):747-755. doi: 10.1111/cid.12958. Epub 2020 Oct 28. PMID: 33112508.
25. Wang F, Fan S, Huang W, Shen Y, Li C, Wu Y. Dynamic navigation for prosthetically driven zygomatic implant placement in extensive maxillary defects: Results of a prospective case series. Clin Implant Dent Relat Res. 2022 Aug;24(4):435-443. doi: 10.1111/cid.13101. Epub 2022 May 23. PMID: 35605154.
26. Shen Y, Dai Q, Tao B, Huang W, Wang F, Lan K, Sun Y, Ling X, Yan L, Wang Y, Wu Y. Real-Time Dynamic Navigation System for the Precise Quad-Zygomatic Implant Placement in a Patient with a Severely Atrophic Maxilla. J Vis Exp. 2021 Oct 18;(176). doi: 10.3791/62489. PMID: 34723941.
